# The MAP Kinase CfPMK1 Is a Key Regulator of Pathogenesis, Development, and Stress Tolerance of *Colletotrichum fructicola*

**DOI:** 10.3389/fmicb.2019.01070

**Published:** 2019-05-21

**Authors:** Xiaofei Liang, Tingyu Wei, Mengyu Cao, Xin Zhang, Wenkui Liu, Yuanyuan Kong, Rong Zhang, Guangyu Sun

**Affiliations:** State Key Laboratory of Crop Stress Biology in Arid Areas and College of Plant Protection, Northwest A&F University, Yangling, China

**Keywords:** *Glomerella*, MAPK, PMK1, *Colletotrichum*, perithecium, sexual reproduction

## Abstract

The Ascomycetes fungus *Colletotrichum fructicola* causes severe diseases on a wide range of crops, fruits, and vegetables. Its pathogenic mechanisms, however, remain poorly understood. Mitogen-activated protein kinases (MAPKs) are conserved regulators of fungal development and pathogenesis. In this study, a Fus3/Kss1-related MAPK from *C. fructicola* was functionally characterized via gene deletion. On potato dextrose agar (PDA) and oatmeal agar media, the *CfPMK1* gene deletion mutants (Δ*CfPMK1*) were slightly reduced in radial growth rate, severely limited in aerial hyphal differentiation and hyphal melanization, and formed deformed perithecia that were smaller in size and more compactly organized relative to wild type. When artificially inoculated on plants, conidia of these mutants failed to differentiate appressoria or penetrate cuticle, and their pathogenicity defect could not be rescued by wounding plant tissue prior to inoculation. On PDA, Δ*CfPMK1* mutants were hypersensitive to osmotic stresses, but were more tolerant to membrane and cell wall stresses. Genetic complementation rescued all phenotypic changes associated with *CfPMK1* gene deletion. Based on GFP fusion expression, CfPMK1 protein accumulation was detected at all life stages, and the accumulation level was higher in nascent appressoria relative to conidia. Overall, this study identified CfPMK1 as a key regulator of appressorium and sexual development, pathogenesis, and stress tolerance in *C. fructicola*.

## Introduction

*Colletotrichum* pathogens cause anthracnose disease on a wide variety of woody and herbaceous crops (Cannon et al., 2012). These pathogens generate huge economic losses and are attractive models for studying the molecular mechanisms underlying fungal hemibiotrophic infection (Crouch et al., 2014). *Colletotrichum fructicola* is a recently established species belonging to the *C. gloeosporioides* species complex. It is distributed worldwide and infects over 50 plant species, among which include important crops such as apple, pear, chili, strawberry, and oil tea (Weir et al., 2012). Diseases caused by *C. fructicola* can be very severe. *Glomerella* leaf spot disease of apple, for instance, causes over 90% defoliation when the environmental conditions are conducive (Wang et al., 2012; Rockenbach et al., 2016). *C. fructicola* is also an important model for studying *Colletotrichum* sexual reproduction. Intense study efforts regarding its sexual reproduction (*Glomerella cingulata*) have led to the proposition of “unbalanced heterothallism” model in the early 20th century (Wheeler, 1954), which explains the complicated mating recognition patterns of *Colletotrichum* isolates (Vaillancourt et al., 2000).

To infect successfully, *Colletotrichum* pathogens develop specialized infectious structures, and produce stage-specific virulence factors (Bailey et al., 1992; O’Connell et al., 2012). Conidial germination leads to the production of a germ tube, which contacts with and adheres to the plant surface, and finally differentiates a melanized appressorium at its terminal end. The melanized appressorium forms a thin penetration peg to penetrate plant cuticle and the epidermal cell wall. Inside the invaded plant cell, the tip of the penetration peg develops thick bulbous biotrophic hyphae without disturbing the integrity of host plasma membrane. In some interactions, these biotrophic hyphae can spread into neighboring plant cells by constricting themselves across narrow plasmodesmata channels (O’Connell et al., 2012). Pathogens generally modify the surface components of biotrophic hyphae to avoid plant recognition or to attenuate plant recognition-triggered immune responses (Oliveira-Garcia and Deising, 2013, 2016; Takahara et al., 2016). At later infection phases, *Colletotrichum* pathogens transit nutrient strategy, and develop thin necrotrophic secondary hyphae. These hyphae secrete toxins and hydrolytic enzymes, the spread of which leads to necrotic symptom development. During *Colletotrichum* infection, the expressions of effector proteins and secondary metabolites are tightly regulated, with many showing stage-specific expression patterns, and playing stage-specific virulence roles (Kleemann et al., 2012; O’Connell et al., 2012).

Diverse signal transduction pathways regulate fungal infection-related morphogenesis, among which the mitogen-activated protein kinase (MAPK) pathway is one of the most well studied (Zhao et al., 2007). MAPKs are a type of serine/threonine kinases occurring among all eukaryotic lineages. These kinases are activated by environmental stimuli via a conserved MAPKKK-MAPKK-MAPK phosphorylation cascade. Activated MAPKs phosphorylate downstream protein targets and regulate gene expression, and in turn, control cellular growth, differentiation, and environmental signal responses. The *Saccharomyces cerevisiae* genome encodes five MAPKs, namely Fus2, Kss1, Slt2, Hog1, and Smk1, which regulate mating pheromone responses, filamentous and invasive growth, cell wall integrity, high-osmolality stress response, and spore wall assembly, respectively. Most filamentous fungi belonging to the Ascomycota phylum contain three MAPKs in their genomes, which are homologous to the *S. cerevisiae* Fus3/Kss1, Slt2, and Hog1, respectively. The functions of these MAPKs have been investigated among different types of phytopathogens (e.g., foliar, root, and vascular pathogens), and in all cases Fus/Kss1 MAPK is indispensable for full virulence (Xu and Hamer, 1996; Lev et al., 1999; Takano et al., 2000; Pietro et al., 2001; Rauyaree et al., 2005; Zhao et al., 2005; Cousin et al., 2006), and for this reason the Fus/Kss1 MAPK has been referred to as the pathogenicity MAPK (PMK). The importance of Slt2 and Hog1 MAPKs in virulence varies among pathosystems and are not as conservatively required for virulence as the Fus/Kss1 MAPK (Zhao et al., 2007; Hamel et al., 2012). The functional importance of Fus/Kss1 MAPK lies in its requirement for both infection-related morphogenesis (appressorium development) and post-invasive colonization. By applying a chemical genetic approach, the *Magnaporthe oryzae* Fus/Kss1 MAPK (PMK1) has recently been demonstrated to activate fungal effector gene expression and to control the constriction of invasive hyphae at plasmodesmata sites (Sakulkoo et al., 2018).

*Colletotrichum fructicola* diseases generate huge economic losses. However, because its identity as a unique species has only recently been established, there is a lack of studies investigating the molecular mechanisms regulating its pathogenicity. In this study, we characterized the biological functions of a Fus/Kss1 MAPK gene, namely *CfPMK1* via gene deletion. Our results demonstrated that CfPMK1 is a key regulator of fungal pathogenesis, development of appressoria and perithecia, as well as stress tolerance.

## Materials and Methods

### Fungal Strains, Culture Conditions, and Nucleic Acid Manipulations

The *C. fructicola* strain 1104-6 was used as a wild type (WT) throughout this work. Both 1104-6 and its derivative mutants were maintained on potato dextrose agar (PDA) medium growing at room temperature. For long-term storage, mycelial plugs from each strain were immersed in 15% glycerol solution and stored at –80°C in the Fungal Laboratory, College of Plant Protection, Northwest A&F University. Conidial production was induced with a PDB shake culture method described previously (Wang et al., 2017). Harvested conidia were washed with sterile distilled water three times, re-suspended in water for further experimental usage. Aerial hyphae scraped from PDA colonies were used for genomic DNA extraction with a modified cetyl trimethylammonium bromide (CTAB) procedure (Tai and Tanksley, 1990). Gene deletion mutants and gene complementation mutants were identified by PCR screening of obtained transformants, which were further confirmed by Southern hybridization, which was performed following kit instructions (DIG-High Prime DNA Labeling and Detection Starter Kit II; Roche, catalog number 11585614910). PCR primers used in this study are listed in Supplementary Table S1.

### *CfPMK1* Sequence Analysis, Gene Deletion, and Complementation

The DNA sequence at the *CfPMK1* gene locus was retrieved from the *C. fructicola* 1104-7 genome based on a BLAST search (GenBank accession number MVNS00000000). Putative protein domains were identified by Interproscan. Sequence alignment and phylogenetic tree construction were performed with MEGA 7.0 (Kumar et al., 2016).

A split-marker strategy was used to generate gene deletion constructs. The 5′ flank of *CfPMK1* ORF, being 1,189 bp in length, was PCR amplified with the primer pair Pmk1LF/Pmk1LRAscI (Supplementary Table S1). The obtained PCR product was purified and digested with *Asc*I; similarly, a 1,095 bp 3′ flank DNA was PCR amplified with the primer pair Pmk1RFNotI/Pmk1RR and digested with *Not*I. The digested flank DNAs were ligated with an hph fragment being released from the pMD19T-Hph vector via *Bsa*I digestion. The ligation product was diluted 10-fold and used as a PCR template for amplifying split marker fragments. The primer pair LFNest/XuHyR was used to amplify a 2,000 bp 5′-flank-Hp fragment, and the primer pair XuYgF/RRNest was used to amplify a 2,246 bp Ph-3′-flank fragment. The obtained 5′-flank-Hp and Ph-3′-flank PCR fragments were purified with a TIANGEN purification kit (catalog number: DP204) and used for protoplast transformation. To generate a gene complementation construct, the primer pair Pmk1-ComF/Pmk1-ComR was used to amplify a DNA fragment, encompassing *CfPMK1* 5′ UTR (2,065 bp in length), and its coding region. The obtained PCR fragment, namely Cf-pmk1, was digested with *Not*I/*Eco*RI and inserted into the *Not*I/*Eco*RI site of pHZ100-GFP vector. pHZ100-GFP contains a promoter-less GFP followed by a tubulin terminator, the insertion of the 3,324 bp Cf-pmk1 fragment into its *Not*I/*Eco*RI site allows for native promoter-driven expression of CfPMK1::eGFP.

Polyethylene glycol (PEG) -mediated protoplast transformation was used for generating gene deletion mutants. PDB inoculated with conidia (final concentration 10^6^/mL) was shake-cultured (25°C, 150 rpm) for 2 days to produce young hyphae. Approximately 0.5 g fungal hyphae were washed with ddH_2_O and 0.8 M KCl, and transferred into a centrifugation tube containing 20 mL protoplast lysis buffer [0.8 M KCl, 10 mg/mL Driselase (Sigma-Aldrich, catalog number D8037), 5 mg/mL lysing enzyme (Sigma-Aldrich, catalog number L1412)]. After lysis for 3 h (30°C, 60 rpm), protoplasts were filtered through MiraCloth (Millipore) and collected by centrifugation (3000 rpm, 10 min). The protoplasts were washed twice with STC buffer (1 M sorbitol, 50 mM Tris–HCl pH 8.0, 50 mM CaCl_2_) and suspended to a final concentration of 10^7^/mL for further usage.

For protoplast transformation, a 20 μL mixture containing 5 μg DNA, 10 mM spermidine, 1 mg/mL heparin sodium was mixed together with 200 μL of protoplasts. After co-incubation on ice for 20 min, PEG8000 was added into the mixture to a final concentration of 60%. Protoplasts were regenerated in TB3 (3 g/L yeast extract, 3 g/L casamino acids, and 20% sucrose) and selected on TB3 containing antibiotics (100 μg/mL hygromycin or 300 μg/mL geneticin).

### Growth, Development, Stress Tolerance, and Virulence Phenotypes

Fungal vegetative growth rates were quantified on PDA and PDA amended with different chemical compounds. 2 mM H_2_O_2_, 2% SDS, and 1,000 μg/mL Congo Red were used to mimic oxidative, membrane, and cell wall stresses, respectively. 0.5 M NaCl and 0.5 M KCl were used to mimic osmotic stress. PDA buffered at pH 3.0 and pH 8.0 were used to test pH stress, for which sterilized 2 × PDA being pre-cooled to 65°C was mixed with an equal volume of sterilized buffer solution. Phosphate–citrate buffer (made up of 0.2 M Na_2_HPO_4_ and 0.1 M citrate) was used for pH 3.0, whereas Sørensen’s phosphate buffer (made up of 0.2 M NaH_2_PO_4_ and 0.2 M Na_2_HPO_4_) was used for pH 8.0.

To examine colony morphology and perithecial development phenotypes, strains were cultured on PDA and OA medium at room temperature (∼25°C). To quantify perithecium density, 18-day old OA cultures were photographed under a dissecting microscope, and perithecial number in individual optical fields (5.2 mm^2^) were counted with a five-point sampling approach (0.25 mm^2^ per point). For each strain, perithecia from six independent optical fields were counted. To quantify perithecium size, 18-day old OA cultures were photographed under a microscope. Diameters of over 70 randomly selected perithecia were measured for each strain. For scanning electron microscopy, PDA agar chunks bearing mycelia and perithecia were fixed with 4% glutaraldehyde in 0.2 M phosphate buffer saline (PBS) (pH 6.8) for 24 h at 4°C, washed with the same buffer for three times, and dehydrated with graded ethanol rinses. The specimens were dried in a vacuum freeze dryer, sputter coated with gold-palladium, and observed with a HITACHI scanning electron microscope (S-3400 N) at 5 kV accelerating voltage. GFP fluorescence was observed under an epifluorescence microscope (Olympus BX51) using FITC/GFP filter.

To examine conidial germination and appressorium development, conidial suspensions (10^5^/mL) were inoculated on onion epidermal peels, and incubated at 25°C for 12 h. Infection structure development was observed with a microscope. To assess pathogenicity variation of strains toward apple leaf, freshly harvested Gala apple leaves were spray-inoculated with conidial suspensions (10^6^/mL) and kept inside a moisture chamber in darkness for 5 days. Inoculated leaves were also sampled at 24 h post inoculation (hpi) to examine appressorium development. Sampled leaves were cleared in ethanol/chloroform (3:1, V/V), containing 0.45% (W/V) trichloroacetic acid, further cleared in chloral hydrate (250 g/100 mL H_2_O), and stained with trypan blue (10 mg/mL in lactophenol) prior to observation. To assess the effect of wounding on infection outcome, pre-wounded Gala apple leaves, and pear fruits (from a local grocery store) were inoculated with mycelial plugs. For leaf wounding, five tiny holes were made by pin-pricking; for fruit wounding, small areas of fruit peel were removed with the combined use of a cork borer, and scalpel. Inoculated leaves and fruits were then kept inside a moisture chamber to promote disease development.

qRT-PCR experiment was performed to compare plant defense reactions triggered by WT and mutant conidial inoculations. Detached Gala leaves were drop-inoculated with ddH_2_O (Mock) or conidial suspension (10^6^/mL), leaves were kept inside a moisture chamber, and harvested at 0 and 12 hpi. RNA extraction, reverse transcription, qRT-PCR amplification, and relative quantifications were performed in the same way as previously described (Liang et al., 2018). Plant defense gene selection and qRT-PCR primer design were based on a previous publication (Vergne et al., 2014), with tubulin alpha-1 gene serving as the internal control.

## Results

### Generating Gene Deletion and Complementation Mutants of *CfPMK1*

**FIGURE 1:**
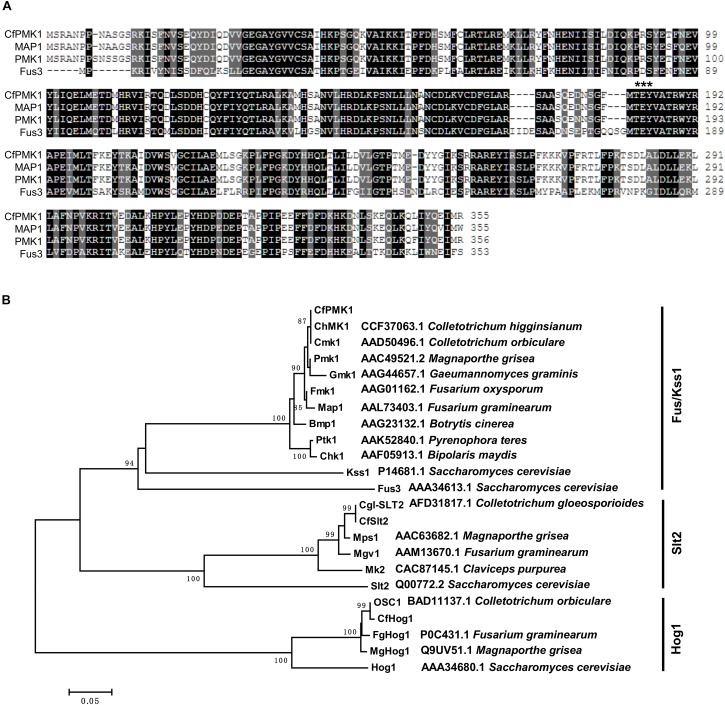
CfPMK1 is a Fus/Kss1 mitogen-activated protein kinase (MAPK). **(A)** Protein sequence alignment of CfPMK1 and MAP1 from *Fusarium graminearum*, PMK1 from *Magnaporthe grisea*, and Fus3 from *Saccharomyces cerevisiae*. Black background indicates identical amino acid residues and gray background indicates similar residues. The “TGY” motif required for kinase activation is marked by asterisk. **(B)** Neighbor-joining phylogenetic tree of CfPMK1, CfSlt2, CfHog1, and homologs from other fungi, numbers at nodes are bootstrap values calculated from 1,000 random replications.

*CfPMK1* was identified as a gene exhibiting high similarity to the *M. oryzae PMK1* gene. Its coding region is 1202 bp long, containing three introns, and encoding a 355-amino acid (aa) protein. The CfPMK1 protein contained a serine/threonine kinase domain (aa 23–311) and a conserved TEY motif (aa 183-185), being putatively required for kinase activation (Figure 1). CfPMK1 displayed a high level of amino acid identity with known Fus3/Kss1 orthologs, such as the *S. cerevisiae* Fus3 (58.4%), the *S. cerevisiae* Kss1 (58.2%), the *M. oryzae* PMK1 (98.6%), the *Fusarium graminearum* Map1 (98.0%), the *Colletotrichum orbiculare* CMK1 (99.4%), and the *C. higginsianum* ChMK1 (100%). A BlastP search identified single copy Slt2 and Hog1 genes from the *C. fructicola* genome, which were highly similar to characterized *Colletotrichum* orthologs, such as Cgl-SLT2 in *C. gloeosporioides* and OSC1 in *C. orbiculare*. Phylogenetically, CfPMK1 and Fus3/Kss1 MAPKs formed a monophyletic clade well separated from the Slt2 and Hog1 clades (Figure 1). These results strongly supported the identity of CfPMK1 as a Fus3/Kss1 MAPK.

**FIGURE 2:**
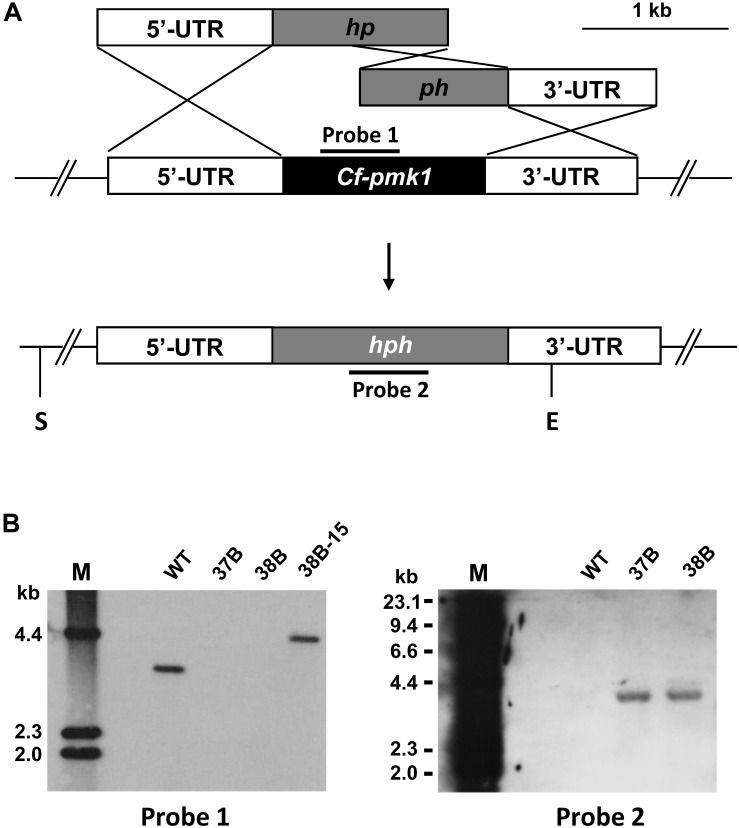
Generating gene deletion mutants of the *CfPMK1* gene. **(A)** *CfPMK1* gene locus and gene replacement constructs. S, *Sal*I; E, *Eco*RV. **(B)** Southern blot of *Sal*I/*Eco*RV digested genomic DNA hybridized with probe 1 (left) and probe 2 (right).

*CfPMK1* gene deletion mutants were generated to investigate its biological functions. Via protoplast-mediated transformation, the wild-type (WT) *CfPMK1* locus was replaced with a hygromycin resistance cassette (Figure 2A). Gene deletion mutants were identified from hygromycin-resistant transformants by PCR screening and Southern hybridization. Two monoconidium-purified mutants, 37B and 38B, were chosen for further phenotypic characterization. The deletion mutant 38B was used for complementation, and one complementation strain named 38B-15 (Δ*CfPMK1/CfPMK1*) was selected for further phenotyping. When *Eco*RV/*Sal*I digested genomic DNAs were probed with the PMK1 fragment amplified with primer pair Pmk1DF/Pmk1DR, the WT 1104-6 produced a 3.6 kb band, the complementation strain 38B-15 produced a 4.3 kb band, yet the gene deletion mutants 37B, and 38B produced no band (Figure 2B, left). When *Eco*RV/*Sal*I digested genomic DNAs were hybridized with the hygromycin phosphotransferase (*hph*) gene, 1104-6 produced no hybridization signal, whereas 37B and 38B produced a 3.9 kb band (Figure 2B, right). Those DNA hybridization signal patterns were in line with expected gene deletion and complementation events in the experimental strains.

### *CfPMK1* Is Required for Normal Colony Morphology and Perithecial Development

*CfPMK1* gene deletion did not affect conidial yield in PDB shake culture (data not shown), but slightly reduced hyphal radial growth rate on PDA. The PDA growth rate of 37B and 38B, 1.33 cm/day, was slightly reduced compared with the 1104-6 (1.53 cm/day) and 38B-15 (1.40 cm/day).

**FIGURE 3:**
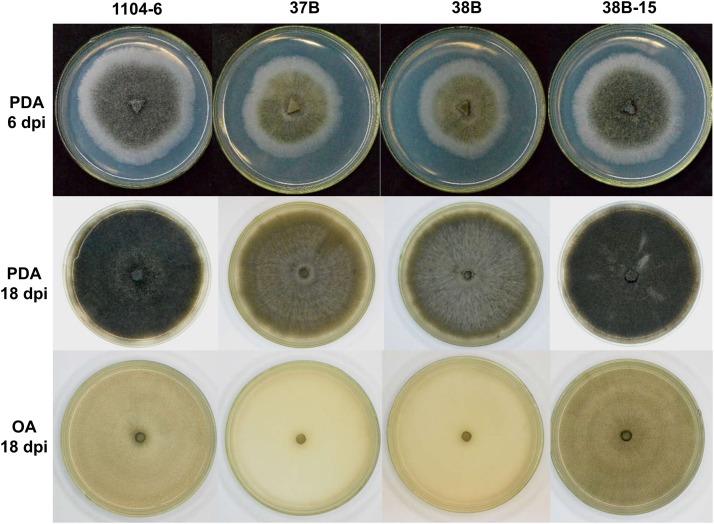
Colony morphologies of wild type (WT, 1104-6), ΔCfPMK1 mutants (37B, 38B), and complementation strain (38B-15) on potato dextrose agar (PDA) and oatmeal agar (OA) media.

*CfPMK1* gene deletion affected colony morphology. At 6 days post inoculation (dpi), PDA colonies of 1104-6 and 38B-15 were similar in morphology, both being melanized, and containing aerial hyphae in the colony center. Colonies became increasingly melanized as they aged (Figure 3). 1104-6 and 38B-15 produced abundant perithecia, visible on OA as scattered black spots (Figure 3). These perithecia rarely produced mature asci, characteristic of the “minus” strain morphology reported by Edgerton (Edgerton, 1914).

**FIGURE 4:**
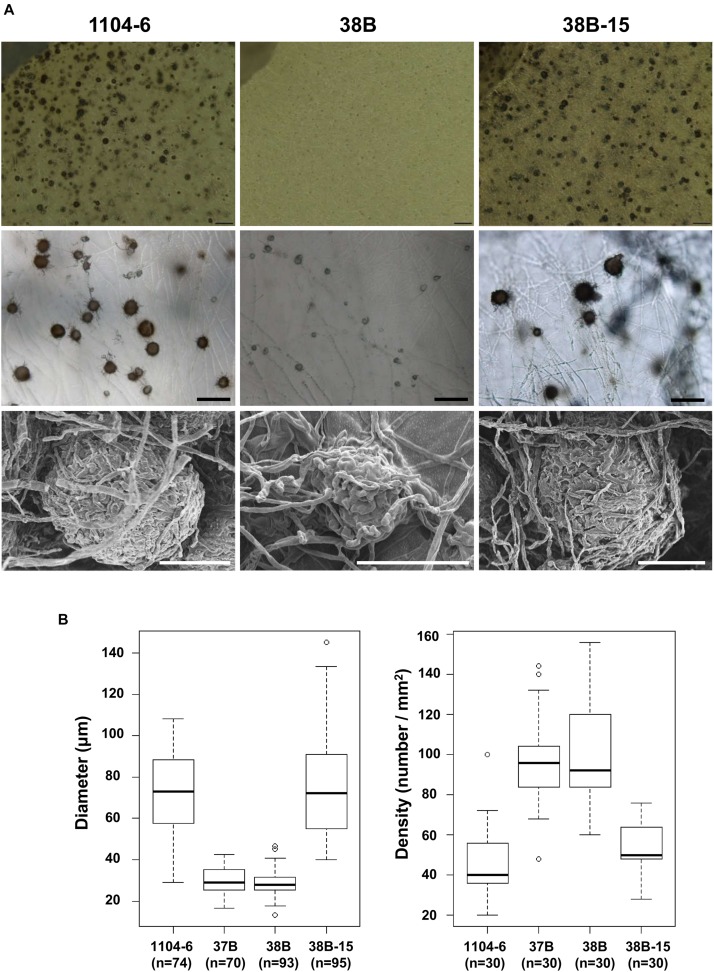
The *CfPMK1* gene is required for normal perithecium development. **(A)** Morphologies of perithecia formed by different strains. Top, dissecting microscopy of OA medium; middle, light microscopy of OA medium; bottom, scanning electron microscopy of PDA medium. The scale bar for light microscopy indicates 200 μm whereas the scale bar for scanning electron microscopy indicates 50 μm. **(B)** Boxplot showing variations of perithecial diameter and density among different strains.

Compared with 1104-6 and 38B-15, colonies of the Δ*CfPMK1* mutants, 37B and 38B, were obviously less melanized, and less fluffy (Figure 3). These Δ*CfPMK1* mutants were also defective in perithecium development (Figures 3, 4). Perithecia formed by 1104-6 and 38B-15 were globose and the outer layers of perithecial walls were heavily melanized. Perithecia formed by 37B and 38B, on the other hand, appeared mis-shaped, more compacted, and lightly melanized (Figure 4A). The average diameters of the perithecia of 37B and 38B were over 2.5-fold smaller than those of 1104-6 and 38B-15 (Figure 4B left), whereas the perithecial densities of 37B and 38B were around twofold higher than 1104-6 and 38B-15 (Figure 4B right). Scanning electron microscopy indicated that the mutants efficiently developed perithecial initials. However, hyphal tip branching of these initials was retarded, which restricted perithecial enlargement (Figure 4A).

### *CfPMK1* Gene Deletion Mutants Are Non-pathogenic and Fail to Form Appressoria

**FIGURE 5:**
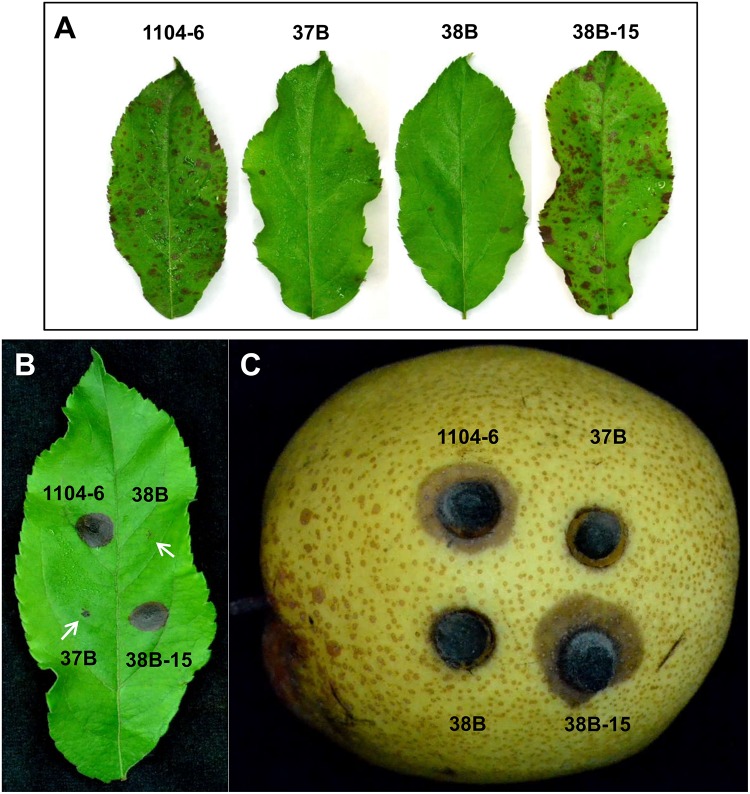
The *CfPMK1* gene is required for pathogenicity. **(A)** Disease symptoms on detached apple leaves (Gala variety) inoculated with conidial suspensions (10^6^/mL) of different strains. Typical leaves were photographed at 5 dpi. **(B)** Disease symptoms on pre-wounded apple leaves (Gala variety) inoculated with mycelial plugs of different strains. Typical leaves were photographed at 3 dpi, arrowheads mark the wound sites for 37B and 38B strains, mycelial plugs were removed away prior to photographing. **(C)** Disease symptom of pre-wounded pear fruit inoculated with mycelial plugs of different strains. Typical leaves were photographed at 2 dpi.

To test the effect of *CfPMK1* deletion on fungal virulence, detached Gala apple leaves were spray-inoculated with conidial suspensions (10^6^/mL), derived from different strains (Figure 5A). At 5 dpi, necrotic lesions were abundant on leaves inoculated with 1104-6 and 38B-15. On the other hand, almost no lesions formed on leaves inoculated with the Δ*CfPMK1* mutants (37B and 38B). Microscopic examination showed that the Δ*CfPMK1* conidia failed to differentiate appressoria, and thus, failed to penetrate cuticle and epidermal cell (detailed in below). To determine whether the Δ*CfPMK1* mutants would be defective in post-invasive colonization, mycelial plugs were inoculated on pre-wounded Gala apple leaves and pre-wounded pear fruits. In both cases, Δ*CfPMK1* mutant inoculation failed to incur lesion formation, whereas inoculation with both 1104-6 and 38B-15 resulted in rapid lesion expansion (Figures 5B,C). Thus, the *CfPMK1* gene is important for post-invasive colonization.

**FIGURE 6:**
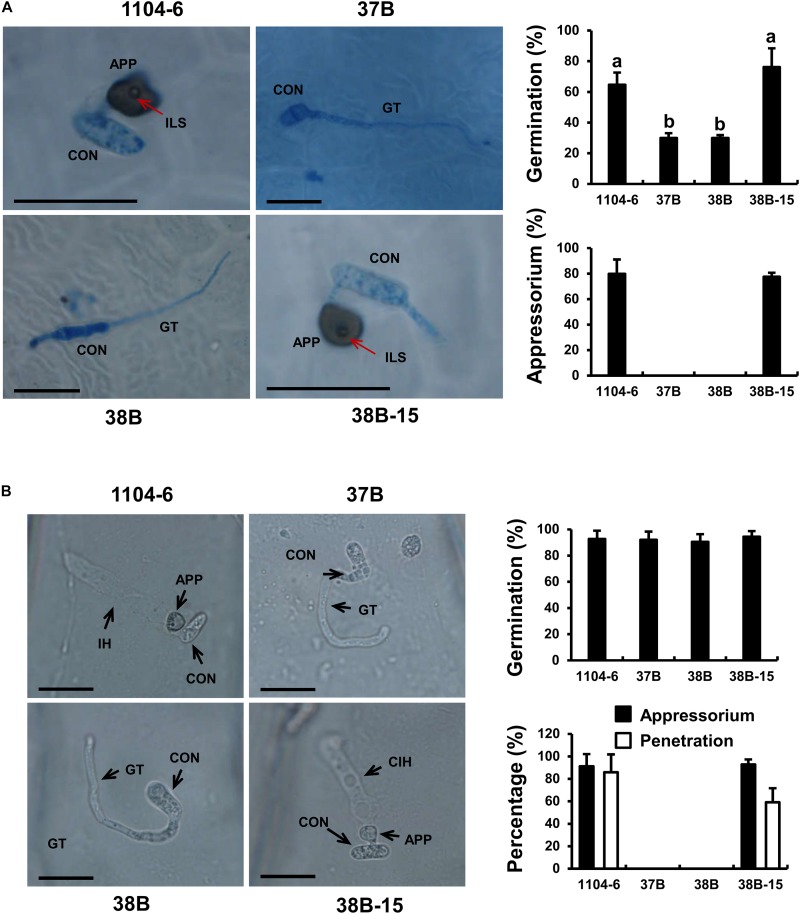
The *CfPMK1* gene is required for appressorium development. **(A)** Conidial germination and appressorium development of different strains on apple leaf surfaces. Apple leaves were sampled at 24 hpi for microscopic observation. “a” and “b” indicate significant difference at *P* = 0.05 according to Tukey’s HSD test following one-way ANOVA analysis. **(B)** Conidial germination and appressorium development of different strains on onion epidermal peels. In **(A,B)**, scale bar indicates 20 μm. CON, conidium; GT, germ tube; APP, appressorium; ILS, internal light spot; IH, infectious hyphae.

The Δ*CfPMK1* conidia (37B and 38B) failed to form appressoria on a range of appressorium-inducive surfaces (e.g., cellophane and cover glass). Quantitative data obtained with apple leaves and onion epidermal peels are presented here in detail. On the apple leaf surface at 1 dpi (Figure 6A), germination rates of Δ*CfPMK1* conidia (37B and 38B) were obviously lower compared with 1104-6 and 38B-15 (29.9% vs. 64.6% and 76.2%, respectively, n≈100 each). Moreover, around 80% of the germinated 1104-6 and 38B-15 conidia developed appressoria, whereas no appressorium formation was observed with those of 37B and 38B (n≈30 each). On detached onion epidermal peels at 14 hpi, conidial germination rates were similar among the four strains, ∼ 90% (n≈100 each). In 1104-6 and 38B-15, 91.1 ± 10.9% and 92.9 ± 4.4% of germinated conidia differentiated appressoria, and 85.8 ± 16.1% and 59.1 ± 12.5% of germinated conidia penetrated and formed infectious hyphae (Figure 6B). Germinated conidia of 37B and 38B, however, never formed appressoria or penetrated the onion epidermis (Figure 6B). These results showed that *CfPMK1* gene is important for appressorium development and penetration.

On apple leaf surface, the Δ*CfPMK1* conidia had reduced germination rate. We tested whether this defect could be triggered by elevated host defense reactions. qRT-PCR was performed to compare the transcriptional induction of apple defense genes at 12 h post inoculation treatments with mock (ddH_2_O), 1104-6 conidia, and 37B conidia (Supplementary Figure S1). Thirteen genes related to callose biosynthesis, redox regulation, PR genes, and markers of the SA, JA, or ET pathways, were assessed. These genes were demonstrated to be responsive to *Erwinia amylovora* infection by a previous publication (Vergne et al., 2014). Compared with mock inoculation at 0 hpi, all 13 genes were up-regulated by the three inoculation treatments, except for LOX in 37B. Compared with mock and 37B treatments, 1104-6 inoculation induced significantly stronger up-regulation of PR2, PR4, WRKY30, ACCO, and EIN3. Compared with mock treatment, both 1104-6 and 37B inoculations induced significantly stronger up-regulation of PR1. However, in no case did we observe a stronger defense gene up-regulation triggered by 37B inoculation relative to 1104-6 inoculation. Thus, we failed to detect a correlation between *CfPMK1* gene deletion and an elevated host defense response at the initial infection phase.

### CfPMK1 Functions in Response to Osmotic and Cell Wall Stresses

**FIGURE 7:**
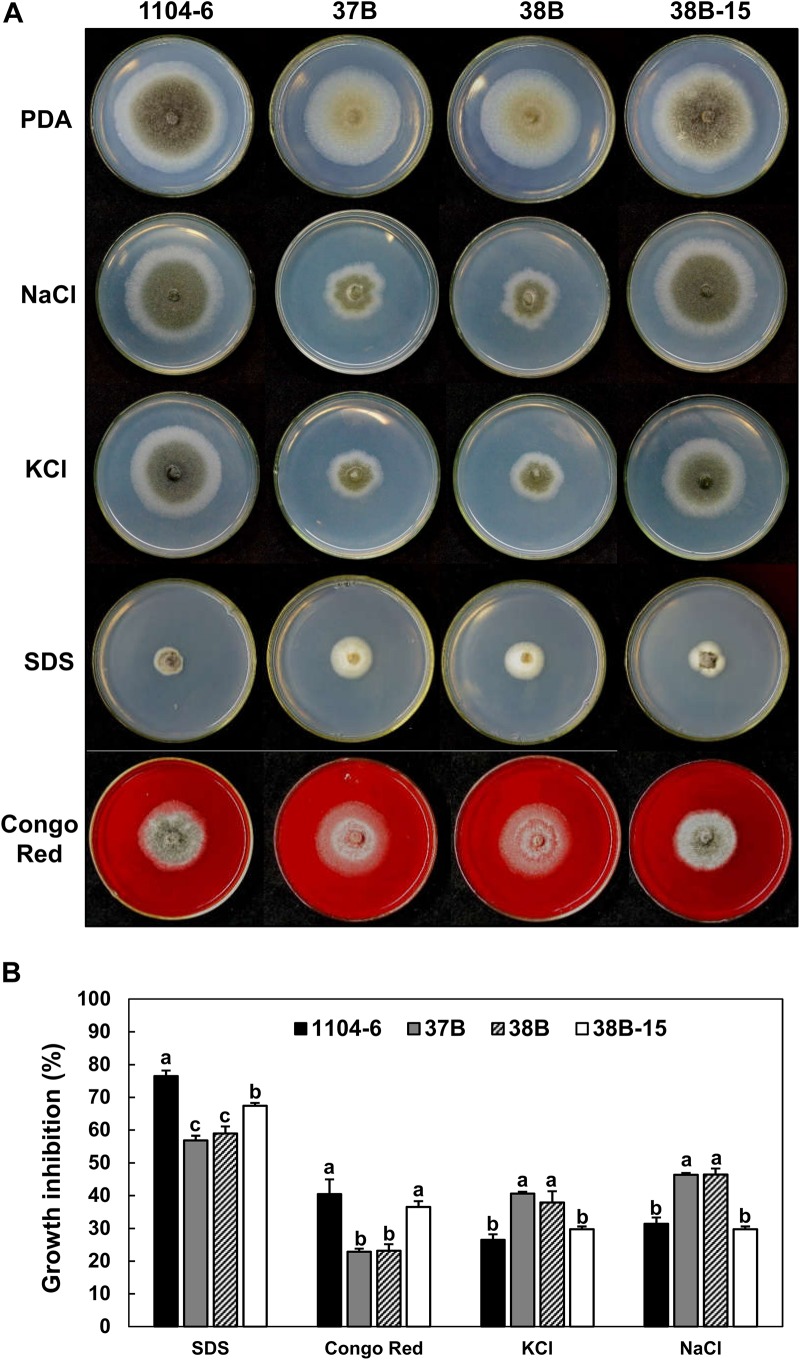
The *CfPMK1* gene functions in stress tolerance. **(A)** Colony morphologies of different strains growing on PDA and PDA amended with different stress compounds. Typical colonies were photographed at 4 dpi. **(B)** Barplot showing variation in growth inhibition of different strains to stress compounds. Growth inhibition was calculated based on growth rates on PDA and PDA supplemented with chemical compounds, bars and error bars represent means ± standard deviations (*n* = 3). Different letters above the error bars indicate significant difference at *P* = 0.05 according to Tukey’s HSD test following one-way ANOVA analysis.

Fungal MAPK genes contribute to stress tolerance. To test the function of CfPMK1, we compared the growth rates of different strains on PDA amended with different stress compounds (Figure 7). Amending PDA with 0.5 M KCl or 0.5 M NaCl strongly inhibited the radial growth of the Δ*CfPMK1* mutants (average inhibition rate, 37.9 and 40.6% for KCl, 46.3% for NaCl), but moderately inhibited the WT and complementation strains (26.5 and 29.7% for KCl, 31.4 and 29.7% for NaCl). In contrast, the Δ*CfPMK1* mutants exhibited faster radial growth compared with the WT and complementation strain on PDA amended with 0.02% SDS or 500 μg/mL Congo Red. The inhibition rates for the mutants were 56.8 and 58.9% for SDS, and 22.9 and 23.2% for Congo Red, whereas the inhibition rates for the WT and complementation strains were 76.5 and 67.4% for SDS, and 40.4 and 36.6% for Congo Red. These results showed that *CfPMK1* gene deletion renders the mutant more sensitive to osmotic stress (KCl and NaCl), but at the same time, enhances tolerance to cell wall and plasma membrane stresses (Congo Red and SDS). *CfPMK1* gene deletion did not affect sensitivity toward low or high pH (PDA medium buffered at 3.0 or 8.0) or oxidative stress (PDA amended with 2.5 mM H_2_O_2_) (data not shown).

### Subcellular Localization of CfPMK1

**FIGURE 8:**
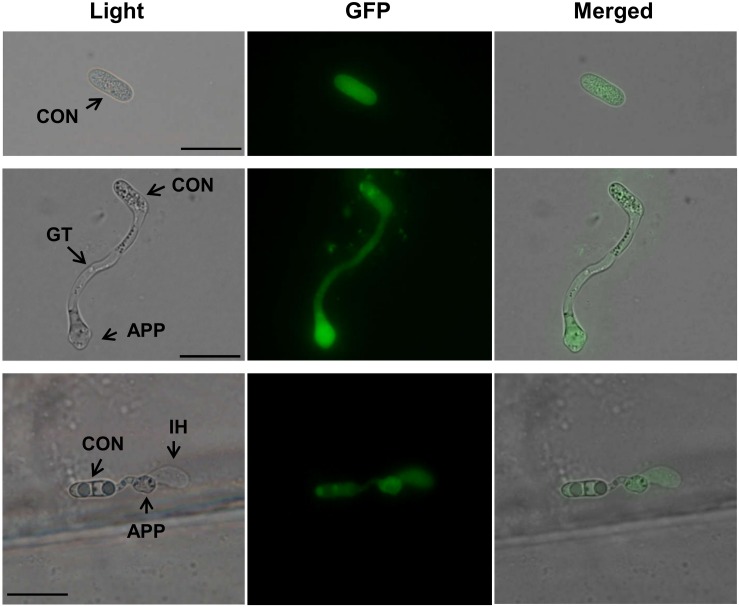
*CfPMK1* gene expression during onion epidermis penetration. Scale bar indicates 20 μm. CON, conidium; APP, appressorium; IH, infectious hyphae.

The complementation strain 38B-15 was used to determine the subcellular localization of CfPMK1, in which CfPMK1::eGFP fusion protein was expressed under the control of the *CfPMK1* native promoter. GFP fluorescence was detected at all stages of fungal life cycle (conidium, germ tube, vegetative hyphae, perithecium, data not shown), and in these structures, the fluorescence signal was distributed in both the cytoplasm and the nucleus. On onion epidermal peels, all infection-related structures (conidium, germ tube, appressorium, and infectious hyphae) emitted GFP fluorescence, and the signal intensity was stronger in nascent appressoria than in conidia and infectious hyphae (Figure 8).

## Discussion

*Colletotrichum fructicola* is an important phytopathogen and Fus/Kss1 MAPK is a conserved pathogenicity regulator in fungi. In this study, we characterized the *C. fructicola* Fus/Kss1 MAPK CfPMK1 and demonstrated that it is a key pathogenicity regulator. We also showed that CfPMK1 regulates development and stress tolerance. While several MAPK pathway genes have been characterized in *C. gloeosporioides* and other *Colletotrichum* pathogens (Takano et al., 2000; Kojima et al., 2002, 2004; Yong et al., 2013; Xiong et al., 2015; Wei et al., 2016; He et al., 2017), this is the first genetic characterization of a *C. fructicola* MAPK gene.

Δ*CfPMK1* conidia were non-pathogenic to apple leaves, which was due to defects in both appressorium differentiation and post-infection colonization, as demonstrated by the inability of Δ*CfPMK1* mutants to infect pre-wounded plant tissues. The critical requirement of Fus/Kss1 MAPK for appressorium formation and post-infection virulence is conserved among a wide range of phytopathogens (Hamel et al., 2012), supporting conserved mechanisms underlying their pathogenic functions. Fus/Kss1 MAPKs regulate downstream targets via target Ser/Thr phosphorylation, and one of the few recognized targets has been the homeodomain transcription factor Ste12 (Wong Sak Hoi and Dumas, 2010). In both *M. oryzae* and *C. orbiculare*, *Ste12* gene disruption mutants develop normal-shaped appressoria, which, however, fail to penetrate (Park et al., 2002; Tsuji et al., 2003). In *M. oryzae*, this penetration defect is associated with a defect in microtubule reorganization and septin ring formation (Park et al., 2004; Dagdas et al., 2012). *Ste12* gene deletion also led to a penetration defect in *C. fructicola* (unpublished data), further supporting the functional conservation between *CfPMK1* and its orthologs. With the aid of a chemical genetics approach, the *M. oryzae* Fus/Kss1 MAPK PMK1 has recently been shown to regulate effector gene expression and the constriction of invasive hyphae across plasmodesmata (Sakulkoo et al., 2018). In the future, a similar approach could be applied to *CfPMK1* to dissect its post-invasive virulence functions.

Within the *Colletotrichum* genus, gene deletion mutants of Fus/Kss1 MAPK have been generated in *C. higginsianum* (Wei et al., 2016), *C. gloeosporioides* (He et al., 2017), *C. orbiculare* (Takano et al., 2000), and *C. truncatum* (Xiong et al., 2015). These mutants have both conserved and have distinctive phenotypes. For instance, all mutant colonies are light-colored, which has been associated with the strong down-regulation of 1,8-DHN melanin biosynthesis gene transcripts. On the other hand, reduced hyphal radial growth rate was observed with mutants in *C. higginsianum*, *C. gloeosporioides*, and *C. orbiculare*, but not with the mutation in *C. truncatum*. In addition, a conidial germination defect was observed with the *C. orbiculare* mutant, but not with mutants from the other three species. In this study, the Δ*CfPMK1* mutants were reduced in growth rate and colony pigmentation, similar to some or all reported *Colletotrichum* mutants. On the other hand, the conidial germination rates of the Δ*CfPMK1* mutants were significantly lower than WT on apple leaf surfaces, despite the finding that their germination rates were similar on onion epidermal peels. Such a plant tissue-dependent germination defect was not observed in the closely related *C. gloeosporioides* lineage (He et al., 2017), supporting the viewpoint that a conserved signal component could have plastic biological effects among closely related lineages, emphasizing the necessity to perform comparative studies. We compared the transcript accumulations of 13 plant defense genes between WT and the Δ*CfPMK1* mutants at 12 hpi, a timepoint corresponding to conidial germination and appressorium formation. None of the genes showed higher expression upon Δ*CfPMK1* inoculation treatment relative to WT treatment, suggesting that Δ*CfPMK1* conidial inoculation does not trigger stronger host defense reactions, which in theory could inhibit conidial germination. We hypothesize that the conidial germination defect of the Δ*CfPMK1* mutant is more likely caused by enhanced sensitivity toward pre-existing conditions (e.g., antimicrobial metabolites on apple leaf surface) rather than by enhanced elicitation of host defense responses.

Compared with WT and complementation strains, the Δ*CfPMK1* mutants were defective in perithecial development, supporting the importance of Fus/Kss1 MAPK for female sexual fruitbody development. In *Neurospora crassa*, all three MAP kinase cascades are required for sexual development and each cascade plays distinct functions during the developmental process (Lichius et al., 2012). The Fus/Kss1 MAPK pathway mutants (Δ*nrc-1*, Δ*mek-2*, and Δ*mak-2*) form densely packed protoperithecia and are defective in trichogyne differentiation. These completely female-sterile mutants, however, are highly male-fertile (Lichius et al., 2012). In the 1950s, McGahen and Wheeler (1951) described the histological events of the *Colletotrichum* (*G. cingulata*) sexual developmental process. Perithecium development is first evidenced by the formation of two lateral initials, which elongate and develop into two coils of cells, an inner coil and an outer coil being distinguishable by propiono-carmine staining intensity. The outer coil develops into a thin peridium surrounding the inner coil. Mating plasmogamy is achieved by directional growth of a copulation hypha and its fusion with the tip cell of the inner coil. The dikaryotic inner coil (also termed the ascogonial coil) produces lateral branches, croziers, and asci. Trichogyne formation, which is common in Ascomycetes fungi, has not been observed with *G. cingulata* (McGahen and Wheeler, 1951). Compared with the WT, perithecia formed by the Δ*CfPMK1* mutants were smaller and more compact, which resembled the phenotypes of Fus/Kss1 MAPK mutants in *N. crassa*. The requirement of CfPMK1 for male and female-fertility during cross-fertilization, however, is unclear. In the future, generating *CfPMK1* gene deletion mutant in a “Plus” type strain being sexually compatible with 1104-6 would be necessary to characterize the function of *CfPMK1* in the mating process.

In filamentous fungi, HOG and SLT2 are the primary types of MAPK regulating stress tolerance. However, the involvement of Fus/Kss1 MAPK in stress response has recently been reported. Deletion of *VmPmk1* in *Valsa mali* renders the mutants more sensitive to oxidative and cell wall stresses (Wu et al., 2017). Deletion of *ChMK1* in *C. higginsianum* increases mutant sensitivity to cell wall inhibitors (Wei et al., 2016). *CgMK1* deletion mutants of *C. gloeosporioides* are more sensitive to osmotic stress (He et al., 2017). In our study, the Δ*CfPMK1* mutants are highly sensitive to high osmolarity stresses (NaCl, KCl) but are more tolerant to cell wall inhibitors (Congo Red, SDS). Both positive and negative crosstalks have been demonstrated among the three MAPK pathways in *Fusarium oxysporum* (David et al., 2017). Likely, Fus/Kss1 MAPK pathway interacts with the other two MAPK pathways in different ways, leading to different stress response phenotypes in different fungi.

In summary, this study identified the *C. fructicola* Fus/Kss1 MAP kinase CfPMK1 as a key regulator of appressorium development, pathogenesis, sexual development, and stress tolerance. The generated *CfPMK1* gene deletion mutant will be a valuable resource for further dissecting the genetic regulation of pathogenesis and sexual reproduction in this important phytopathogen.

## Author Contributions

XL, RZ, and GS conceived and designed the study. XL, TW, MC, WL, XZ, and YK performed the experiments. XL wrote the manuscript. RZ and GS revised the manuscript.

## Conflict of Interest Statement

The authors declare that the research was conducted in the absence of any commercial or financial relationships that could be construed as a potential conflict of interest.
