# Supplementary material for: The MAP Kinase CfPMK1 Is a Key Regulator of Pathogenesis, Development, and Stress Tolerance of Colletotrichum fructicola
Source: Front Microbiol. 2019 May 21;10:1070. doi: 10.3389/fmicb.2019.01070 (PMC6536633; doi:10.3389/fmicb.2019.01070)
Supplement: Supplementary file 1 [file Data_Sheet_1.docx]

**Table S1. Primers used in this study**

| **Name** | **Sequence (5’-3’)** |
| --- | --- |
| Pmk1DF | ACCACTCCATGTTCTGTCTCCGTAC |
| Pmk1DR | CTTTCCGCTAAGCATTTCCGCCAAG |
| Pmk1LF | CTCGTGTTACTGCTGCACCGTTCAC |
| Pmk1RR | GGCGAGAAGTTTGGTGTCTTGGGTG |
| Pmk1LFNest | TTTGCTCCTGCCATCTTCTGGAAGC |
| Pmk1RRNest | TAGACACGGCGAAACGGAGCGTATG |
| Xu855R | GCTGATCTGACCAGTTGC |
| Pmk1LRAscI | GCATTGGCGCGCCAGGTGTTGTCGAGCAGGTAGTTATC |
| Pmk1RFNotI | ATAAGAATGCGGCCGCAAAGGGCTGTCGTCGGGACTTGTTG |
| Xu866F | GTCGATGCGACGCAATCGT |
| XuHyR | AAATTGCCGTCAACCAAGCTCTGATAG |
| XuYgF | TTTCAGCTTCGATGTAGGAGGGCG |
| Pmk1-ComF-NotI | ACCGCGGTGGCGGCCGCGACGATTCTCCTTGTTACCAGCCTC |
| Pmk1-ComR | GCTCACCCTATCGAATTCCCGCATAATCTCCTGGTAAATCAAC |


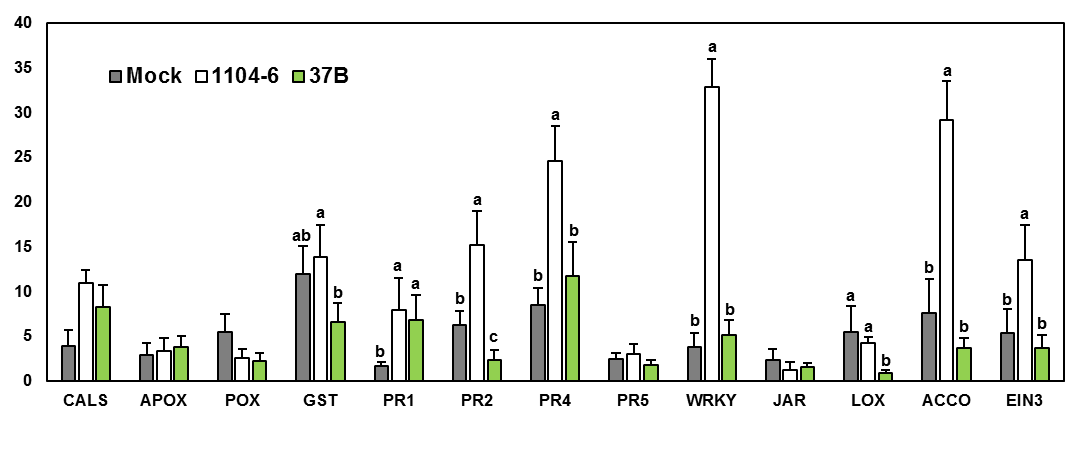


**Figure S1. Expression of apple defense genes in response to different types of challenging inoculations at 12 hpi**. Fold changes relative to mock inoculation at 0 hpi were plotted and error bars were calculated from three independent replicates. For each gene, bars under different letters were significantly different at *P* < 0.05 based on pairwise permutation test. CALS: callose synthase; APOX: ascorbate peroxidase; POX: peroxidase; GST: glutathione S-transferase; WRKY: WRKY transcription factor 30; JAR: jasmonate resistant 1; LOX: lipoxygenase AtLOX2; ACCO: 1-aminocyclopropene-1-carboxylase oxidase; EIN3: EIN3-binding F box protein 1. Tubulin alpha-1 gene was used as the internal control. Please refer to the work by Vergne et al (2014) for GenBank accessions and qRT-PCR primers of the characterized genes.

**Reference**

Vergne, E., de Bernonville, T.D., Dupuis, F., Sourice, S., Cournol, R., Berthelot, P. et al. (2014). Membrane-targeted HrpNEa can modulate apple defense gene expression. *Mol. Plant Microbe Interact.* 27, 125-135. doi: 10.1094/MPMI-10-13-0305-R.
